# Semi-automated workflow for molecular pair analysis and QSAR-assisted transformation space expansion

**DOI:** 10.1186/s13321-021-00564-6

**Published:** 2021-11-13

**Authors:** Zi-Yi Yang, Li Fu, Ai-Ping Lu, Shao Liu, Ting-Jun Hou, Dong-Sheng Cao

**Affiliations:** 1 Xiangya School of Pharmaceutical Sciences, Central South University, Changsha, 410013 Hunan People’s Republic of China; 2 Hunan Key Laboratory of Diagnostic and Therapeutic Drug Research for Chronic Diseases, Changsha, 410013 Hunan China; 3 Institute for Advancing Translational Medicine in Bone & Joint Diseases, School of Chinese Medicine, Hong Kong Baptist University, Hong Kong, 999077 SAR People’s Republic of China; 4 Department of Pharmacy, Xiangya Hospital, Central South University, Changsha, 410008 Hunan People’s Republic of China; 5 Hangzhou Institute of Innovative Medicine, College of Pharmaceutical Sciences, Zhejiang University, Hangzhou, 310058 Zhejiang People’s Republic of China

**Keywords:** MMPA, QSAR, MMPA-by-QSAR pipeline, Lead optimization, Medicinal chemical rule

## Abstract

In the process of drug discovery, the optimization of lead compounds has always been a challenge faced by pharmaceutical chemists. Matched molecular pair analysis (MMPA), a promising tool to efficiently extract and summarize the relationship between structural transformation and property change, is suitable for local structural optimization tasks. Especially, the integration of MMPA with QSAR modeling can further strengthen the utility of MMPA in molecular optimization navigation. In this study, a new semi-automated procedure based on KNIME was developed to support MMPA on both large- and small-scale datasets, including molecular preparation, QSAR model construction, applicability domain evaluation, and MMP calculation and application. Two examples covering regression and classification tasks were provided to gain a better understanding of the importance of MMPA, which has also shown the reliability and utility of this MMPA-by-QSAR pipeline.

**Supplementary Information:**

The online version contains supplementary material available at 10.1186/s13321-021-00564-6.

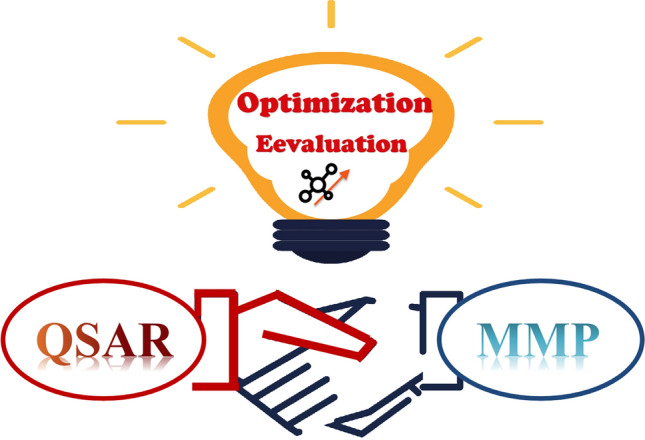

## Introduction

The discovery of drug candidates capable of blocking or activating the desired target proteins involves extensive virtual and experimental screening that accounts for 30–40% of the total time invested in drug development [1]. Given the difficulty of directly finding an optimal drug candidate with desirable therapeutic potency, and absorption, distribution, metabolism and elimination (ADME) and toxicity profile [2], the success of a drug discovery campaign is strongly affected by the efficiency of lead optimization. Traditionally, lead optimization largely relies on heuristic approaches adopted by medicinal chemists, who draw inspiration from their limited experience and synthetic guidelines [3]. Recently, the development of machine learning (ML) methods has enabled the application of deep learning (DL) techniques to lead optimization [4, 5]. Some novel DL algorithms such as variational autoencoders (VAE), recurrent neural networks (RNN), generative adversarial networks (GAN), and graph convolutional networks (GNN) have been utilized to generate novel molecules and optimize their ADMET properties and binding affinity [6–8]. However, the limitations related to interpretability and the optimality of multiple parameters still impede efficient lead optimization [9].

The molecular matched pair (MMP) approach, first proposed by Kenny and Sadowski in 2005, has rapidly become a popular method for the extraction of medicinal chemistry knowledge from large compound/property databases, which can be used in a variety of practical applications, such as compound optimization [10, 11]. An MMP is generally defined as a pair of compounds that can be interconverted by a well-defined chemical transformation at a single site, with the change between the pair elements referring to the transformation, and the invariant feature referring to the context [12]. The systematic extraction and summarization of the MMPs from a large chemical database possess analytical and generative characteristics, which is called matched molecular pair analysis (MMPA) [13, 14]. Compared with DL models, the MMP approach directly deals with measured chemical data and provides a clear interpretation of the results. Moreover, this method allows researchers to directly and easily extract/summarize information from chemical data and thus provides a wide range of functions, including suggestions on what compound should be prepared next, compound property prediction, identification of cases where structural changes have minimal effects on key properties (e.g., bioisosteres), and the simple deepening of our understanding of the links between biology and chemistry [15–18]. Finally, MMPA focuses on local structural transformations rather than the whole molecule and is therefore more suitable for optimization tasks [13].

The fundamental hypothesis of the MMP approach is that a particular change of pharmaceutical properties is contributed from a small structural change. However, in practice, substructural changes are more complex, e.g., the distribution of property changes with respect to transformation rules is often nearly symmetrical and centered at or near zero, which results in a similar likelihood of causing potency gains or losses [19]. Therefore, the inclusion of statistical tests is of utmost importance for further MMP evaluation because it helps to ensure the efficiency and accuracy of MMPA in molecular optimization [20–22]. The application of statistical significance brings the benefit of reduced variation and enhanced credibility of MMPA. However, such measure also proposes the necessary request for dataset size. Unfortunately, most experimental datasets used in drug research are small-scale, which has greatly restricted the scope and utility of chemical transformation mining. To better tackle the limited experimental data problem in MMPA, the MMPA-by-QSAR paradigm was proposed. In this paradigm, QSAR modeling is firstly employed to make predictions for unlabeled data, and MMPA is subsequently used for chemical transformation analysis based on the predicted activities/properties of compounds [23, 24]. The results showed that a large number of useful transformations can be detected by the MMPA-by-QSAR paradigm for driving molecular optimization based on accurate QSAR model predictions. Recently, our research group has taken logD7.4 as an example to show how accurate the predicted results can be gained through the appropriate application of consensus QSAR modeling and applicability domain evaluation [25]. A comparison of the magnitude and directionality of the rules derived from the predicted data with those derived from the experimental data revealed that the mixed data covering credible predicted data and experimental data allow one to generate more design ideas without introducing much noise [25]. Considering the utility of MMPA in molecular optimization, several tools have been developed for MMP construction and aggregation. In 2018, Dalke et al. presented an open-source MMP platform called mmpdb, which applies a fragment-and-index engine with the use of fingerprints for environment capturing [26]. In 2020, Lumley et al. developed the LillyMol toolkit which includes the methods for aggregating MMPs into summarized transformations [27]. However, the above MMP calculation tools are quite difficult to be used for researchers with poor programming background, thus impeding the achievement of more meaningful MMPA. More importantly, none of the above tools is specifically designed for MMPA-by-QSAR manipulation, which has largely limited the chemical exploration of experimental datasets, especially for small datasets.

Herein, we developed a new semi-automated pipeline based on the KNIME platform to support chemical transformation mining for either large- or small-scale experimental datasets. For large datasets, the integral compilation of MMP calculation, chemical context clustering, statistical test and transformation application can aid an understanding of the structural changes that drive the optimization of key pharmacological properties. More importantly, this work also accomplishes a comprehensive MMPA-by-QSAR pipeline for small datasets, including molecular preparation, QSAR model construction, applicability domain evaluation, MMP calculation, and transformation generation and application. The combination of QSAR and MMPA enables not only the discovery of new transformations but also the amplification of existing ones by providing more evidence of the observed effects. It is believed that the reasonable application of this pipeline can be beneficial to the automated optimization of suboptimal molecular properties during the early stages of drug discovery and development.

## Materials and methods

### Computational tools

This study was performed using the open-source KNIME v. 4.1.2 software available free of charge at https://www.knime.com [28]. The related extensions were automatically installed after KNIME import. Before the above program was executed, the Python environment and R path were correctly configured, as seen in “File>>>Preferences>>>KNIME>>>Python” and “File>>>Preferences>>>KNIME>>>R,” respectively. The R version should be higher than 3.6.0. We used Python 3, and the downloaded RDKit, pandas, sklearn, numpy, matplotlib and Scopy (https://github.com/kotori-y/Scopy) modules [29]. More details about the dependencies in the pipeline are summarized in Additional file 4: Table S1.

### Workflow description

**Fig. 1:**
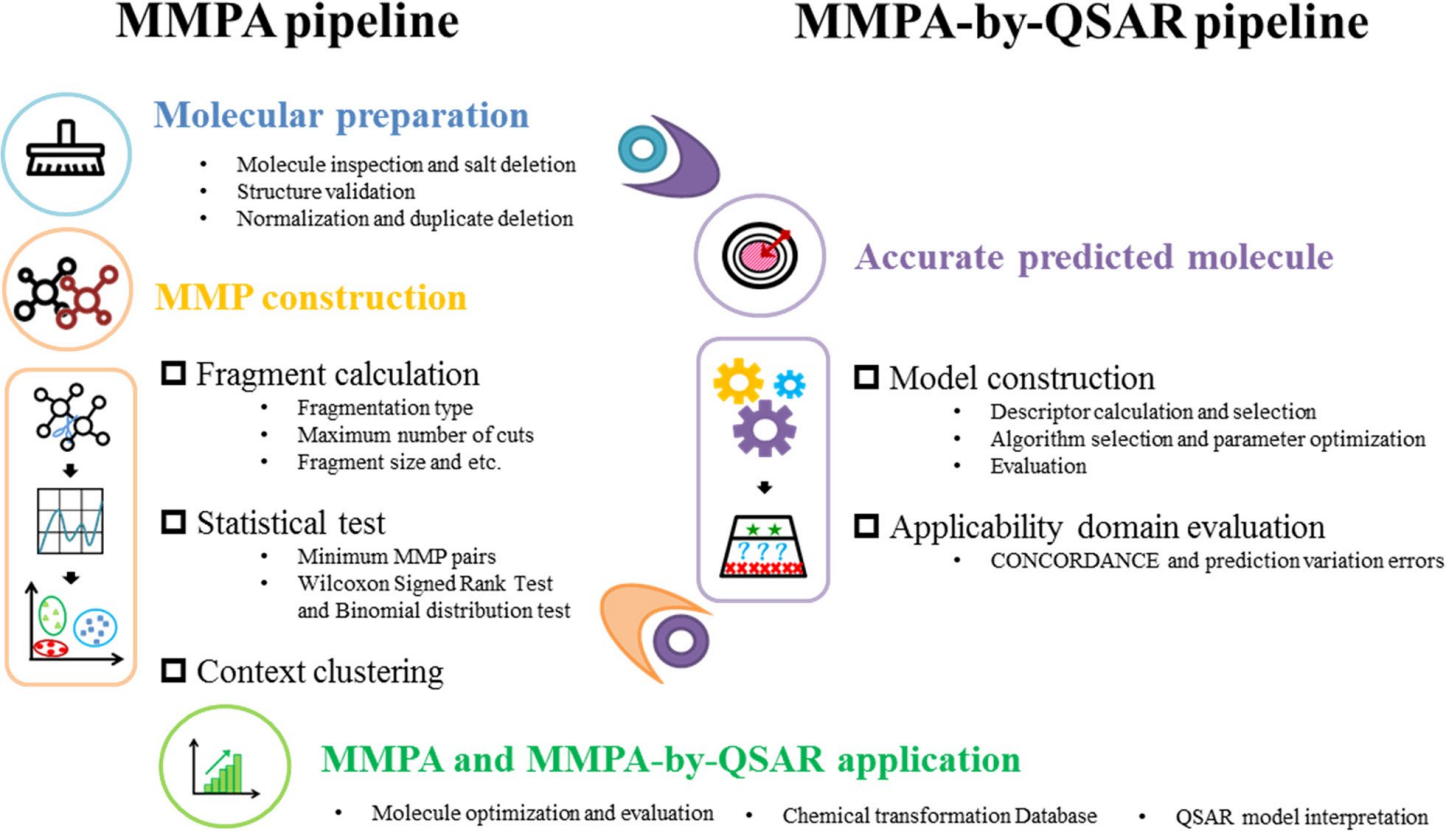
Representation of MMPA and QSAR-assisted-MMPA pipeline

The procedure described below was implemented as a KNIME workflow. To benefit the utilization, the guidance for users on how to install and use the workflow is provided in Additional file 3. This pipeline is called “semi-automated” because the most parts can be achieved with a click of mouse. However, expert judgment and manual inspection are needed and even necessary in the whole process, since some errors are obvious to a human, but are still not obvious for computers. Therefore, a final manual intervention is required to check the presence of errors that cannot be identified by a completely automated procedure. The whole workflow includes three main parts, namely data preparation, model construction and evaluation, and MMP calculation (Fig. 1).

### Data preparation

The checking and preparation of molecular structures are the necessary prerequisites for the subsequent structural analysis. For QSAR model derivation, data quality is extremely important, as it strongly affects the robustness of and predictive power of the final model [30]. For MMPA, the compounds used for analysis must be in a consistent salt, charge, and tautomeric state [31]. Considering the requirements of QSAR analysis and MMPA, the molecular preparation module was designed as follows: (1) inspection of broken bonds, dummy atoms, and charges; (2) salt and mixture removal; (3) normalization of functional groups to a consistent format and tautomer enumeration; (4) labeling of uncommon element and chirality information; and (5) normalization and duplicate deletion [32]. It is believed that, through the systematic data curation, the analysis and summary of chemical transformation will be more reliable.

### Model construction and evaluation

The construction of accurate and credible prediction models is of utmost importance for MMPA-by-QSAR. To achieve the expansion of credible prediction results, this pipeline provides a convenient and comprehensive module for model construction and evaluation, including descriptor calculation and selection, model construction and evaluation, and the selection of accurate prediction results.

For descriptor calculation, the MMPA-by-QSAR pipeline provides 17 frequently used descriptors and fingerprints, including MOE2D, RDKit, Morgan fingerprints, etc. The QSAR models based on these descriptors and fingerprints that represent comprehensive structural and physicochemical information tend to have good predictive performance. In addition, this pipeline also supports the calculation of two types of molecular scaffolds, namely Murcko scaffolds and carbon skeletons, for the exploration of chemical diversity and further data grouping [32, 33]. To remove irrelevant variables, the feature selection process is provided as the following steps: (1) the descriptors with a variance of zero or close to zero are deleted; (2) if the correlation coefficient between two descriptors exceeds 0.95, only one descriptor is selected; (3) the recursive feature elimination algorithm is used for variable selection [34]. Such detailed settings allow both efficient molecular feature extraction and credible model construction.

In addition to descriptor calculation, the choice of an appropriate ML algorithm is also important for the effectiveness of prediction models. Based on our previous experiences, four effective ML algorithms, namely random forest (RF), extreme gradient boosting (XGBoost), support vector machine (SVM), and gradient boosting (GB), are provided in this pipeline for model construction [35–37]. According to our previous studies, a consensus model constructed by averaging the outputs of multiple individual models is recommended for the final predictions in this pipeline [38–41]. Considering the importance of model hyper-parameters, the MMPA-by-QSAR pipeline uses the grid search method and a validation set to optimize model hyper-parameters. To benefit the efficiency of model construction, the common scopes of important parameters of different algorithms were summarized in Additional file 3.

To ensure that the prediction models are qualified for MMPA-by-QSAR, fivefold cross-validation and test sets were used for evaluation. For classification models, the evaluation statistics include overall prediction accuracy, prediction accuracy of the positive set (sensitivity), prediction accuracy of the negative set (specificity), F-index, precision and recall (Table 1). In addition, receiver operating characteristic curve and area under the receiver operating characteristic curve (AUC) were used to evaluate the comprehensive performance of classification models. For regression models, three main statistical parameters, including squared correlation coefficient (*Q*^2^/R^2^), root mean squared error (RMSE) and mean absolute error (MAE), were applied to evaluate models.

**Table 1:** The statistical parameters of model prediction performance

Category	Parameters	Definition	Meaning
Classification prediction models	True positive (TP)	Real label = 1 and predicted label = 1	Number of correctly classified positive results
	True negative (TN)	Real label = 0 and predicted label = 0	Number of correctly classified negative results
	False positive (FP)	Real label = 0 and predicted label = 1	Number of misclassified positive results
	False negative (FN)	Real label = 1 and predicted label = 0	Number of misclassified negative results
	Accuracy (ACC)	ACC = (TP + TN)/(TP + TN + FP + FN)	Overall prediction accuracy
	Sensitivity (SE)	SE = TP/(TP + FN)	Prediction accuracy of the positive set
	Specificity (SP)	SP = TN/(TN + FP)	Prediction accuracy of the negative set
	Precision	Precision = TP/(TP + FP)	Efficiency of positive results prediction
	Recall	Recall = TP/(TP + FN)	Coverage of positive results prediction
	Index F (F1)	F1 = 2Precison * Recall/(Precision + Recall)	Evaluation of the comprehensive performance of the models
	Receiver operating characteristic (ROC) curve area under the Roc curve (AUC)	The probability that a randomly chosen positive example is ranked higher than a randomly chosen negative example	The performance of the classification model as its discrimination threshold is varied
Regression prediction model	Squared correlation coefficient (Q^2^/R^2^)	Q^2^/R^2^ = $1 - \frac{{\mathop \sum \nolimits_{i = 1}^{m} \left( {y_{i} - \widehat{{y_{i} }}} \right)^{2} }}{{\mathop \sum \nolimits_{i = 1}^{m} \left( {y_{i} - \overline{y}_{i} } \right)^{2} }}$	Squared correlation coefficient
	Mean absolute error of cross validation (MAE)	MAE = $\frac{1}{m}\mathop \sum \limits_{i = 1}^{m} \left( {y_{i} - \widehat{{y_{i} }}} \right)^{2}$	Mean absolute error of cross validation
	Root mean squared error (RMSE)	RMSE = $\surd \left( {{\text{MAE}}} \right)$	Root mean squared error

Except integral model evaluation, detection of the accurate results for the predicted molecules is even more important, since the QSAR-based prediction of pharmacological or physicochemical properties is of limited value without an estimated model applicability domain (AD). Therefore, AD evaluation was provided in this pipeline for accurate prediction selection and credible expanded dataset construction. CONCORDANCE, a parameter which reflects the number of models that provide the same prediction result of the current models to consensus model, was mainly used for classification prediction accuracy assessment (Formula 1) [42].

1 $$CONCORDANCE = \mathop \sum \limits_{i = 1}^{N} eq\left( {y\left( M \right),y_{i} \left( M \right)} \right),$$

where y(M) and y_i_(M) are the predicted results of compound *M*, given by the consensus model and single models, *N* is the number of all models (includes the consensus model), and *eq* is equality indicator (equal to 1 if the arguments are equal and to 0 otherwise). In addition, the predicted score of the consensus model was also used as a supplement, since compounds with higher (or lower) prediction scores are more likely to be positive-label data (for negative-label data) [38, 40].

For regression models, the standard derivation of the ensemble members (Ensemble_SD) was used for the model AD evaluation, which is defined as follows:

2 $${\text{Ensemble}}\_SD = \sqrt {\frac{1}{N - 1}\mathop \sum \limits_{i = 1}^{N} \left( {X_{i} - \overline{X}} \right)^{2} } ,$$

where *X*_*i*_ and $\overline{X}$ are the predicted results of a model and the final consensus model, respectively, and *N* is the number of models. The standard deviation of the ensemble members' output can be viewed as a way to characterize the reliability of predictions in regression problems [43]. Therefore, Ensemble_SD is a measure of the variability of the prediction, where the less variation is more likely to get more accurate prediction [44, 45].

However, it should be noted that though the application of the above methods can be beneficial for discriminating reliable prediction results, the current state of AD evaluation is still under exploration. Therefore, it is suggested to use a stringent threshold for AD coverage to detect accurately predicted molecules to avoid noise introduction. Only the predicted compounds meeting the stringent AD requirement can be used for the construction of reliable chemical transformation rules.

### MMP analysis

In the present pipeline, MMPs were generated using an implementation of the Hussain and Rea algorithm, which identifies shared substructures by fragmenting each molecule and then storing and indexing all enumerated fragments in an inverted-file-like structure [46]. Several parameters can be adjusted during the fragmenting process, such as fragmentation type, hydrogen manipulation, maximum number of cuts, maximum number of heavy atoms in fragments, and the ratio of changing to unchanging parts (for details, see Additional file 3), to meet the need of different research tasks. To ensure the credibility of the compiled chemical transformation, the requirement for the minimum number of MMPs and statistical significance tests were also integrated into this pipeline. The Wilcoxon signed rank test for continuous variables (alpha = 0.05) and the binomial distribution test for discrete variables (probability of success = 0.5, alpha = 0.05) were used to evaluate the statistical significance of continuous and binomial values, respectively. In this study, the term of “rule” refers to the chemical transformation leading to a change in a pharmacological or physiochemical endpoint which passes the statistical significance test with enough MMPs. As the rules are generalized over many compound pairs, the context information of a specific MMP is important for detecting the specificity and generalizability of structural changes [12]. Therefore, in this pipeline, the Morgan fingerprint of the entire molecular context was used for the corresponding clustering, which enables the detection of the chemotype diversity of the summarized rules. Actually, it is suggested that at least five clusters are needed for the generality of a chemical transformation [47]. After MMP calculation and rule compilation, the final rules can be applied to optimize the properties of initial molecules, and the optimized molecules can be further evaluated according to the drug-likeness index and substructural rules [29, 48]. The comprehensive workflow of MMP calculation and application has benefited the efficiency of chemical transformation discovery that can be used for the property optimization of lead molecules.

## Results and discussion

To better understand the utility and advantages of the MMPA-by-QSAR pipeline, we selected the MMPA of logP and human hERG (including both regression and classification prediction models) as two examples for further explanation [49, 50]. Two commercial compound libraries, ChemBridge (1,595,088 molecules) and ChemDiv (1,962,494 molecules), were selected as the external datasets for model prediction.

### MMPA of logP dataset

logP, representing molecular lipophilicity, significantly influences drug potency, ADME and toxicity. Compounds with high lipophilicity are more likely to bind multiple targets, which increases the probability of high promiscuity [48]. Whereas compounds with low lipophilicity are more likely to exhibit problematic permeability and renal clearance. Therefore, the optimal lipophilicity range is highlighted in drug design and optimization phases. For a better understanding of the effective substructural transformation rules of logP, 16,146 compounds with experimental logP values were collected from ADMETlab [49, 50]. Two molecular representations (MACCS and MOE2D descriptors) and four ML algorithms were used to construct the logP prediction models. The consensus model was finally constructed by averaging the prediction values of eight individual models (based on the combination of four ML algorithms and two sets of molecular descriptors). To verify the reliability and predictive ability of the prediction models, according to the calculated chemical scaffolds, all the compounds were divided into the training set (12,916 compounds, 80%), validation set (1615 compounds, 10%), and test set (1615 compounds, 10%), which were used for model construction, hyper-parameter optimization, and model evaluation, respectively. The statistical results of the 10 fivefold cross-validations and test set predictions are summarized in Table 2.

**Table 2:** Performance of the logP prediction models derived from different combinations of ML algorithms and descriptor sets

	Fivefold cross-validation			Test set		
	Q^2a^	MAE_cv_^a^	RMSE_cv_^a^	R_test_^2a^	MAE_test_^a^	RMSE_test_^a^
MACCS						
GB	0.931	0.338	0.471	0.950	0.294	0.410
RF	0.873	0.470	0.640	0.907	0.405	0.561
SVM	0.882	0.458	0.616	0.905	0.419	0.567
XGBoost	0.931	0.333	0.470	0.955	0.279	0.388
MOE2D						
GB	0.944	0.292	0.423	0.954	0.264	0.395
RF	0.922	0.358	0.501	0.937	0.320	0.461
SVM	0.922	0.346	0.500	0.937	0.309	0.464
XGBoost	0.946	0.289	0.417	0.956	0.263	0.386
Consensus model	0.943	0.304	0.426	0.957	0.267	0.382

^a^The squared correlation coefficient of the cross-validation and test set prediction (Q^2^ and R_test_^2^), the mean absolute error of the cross-validation and test set prediction (MAE_cv_ and MAE_test_), and the root mean squared error of the cross-validation and test set prediction (RMSE_cv_ and RMSE_test_)

As shown in Table 2, all the nine ML models performed well for both the fivefold cross-validation and test sets, featuring average Q^2^ and R_test_^2^ of 0.921 and 0.939, respectively, and thus having high prediction capability. Among the nine ML models, the consensus model showed the best prediction ability, as revealed by its high Q^2^ (0.943) and R_test_^2^ (0.957), and was therefore applied to predict the unknown molecules in the two commercial compound libraries.

**Fig. 2:**
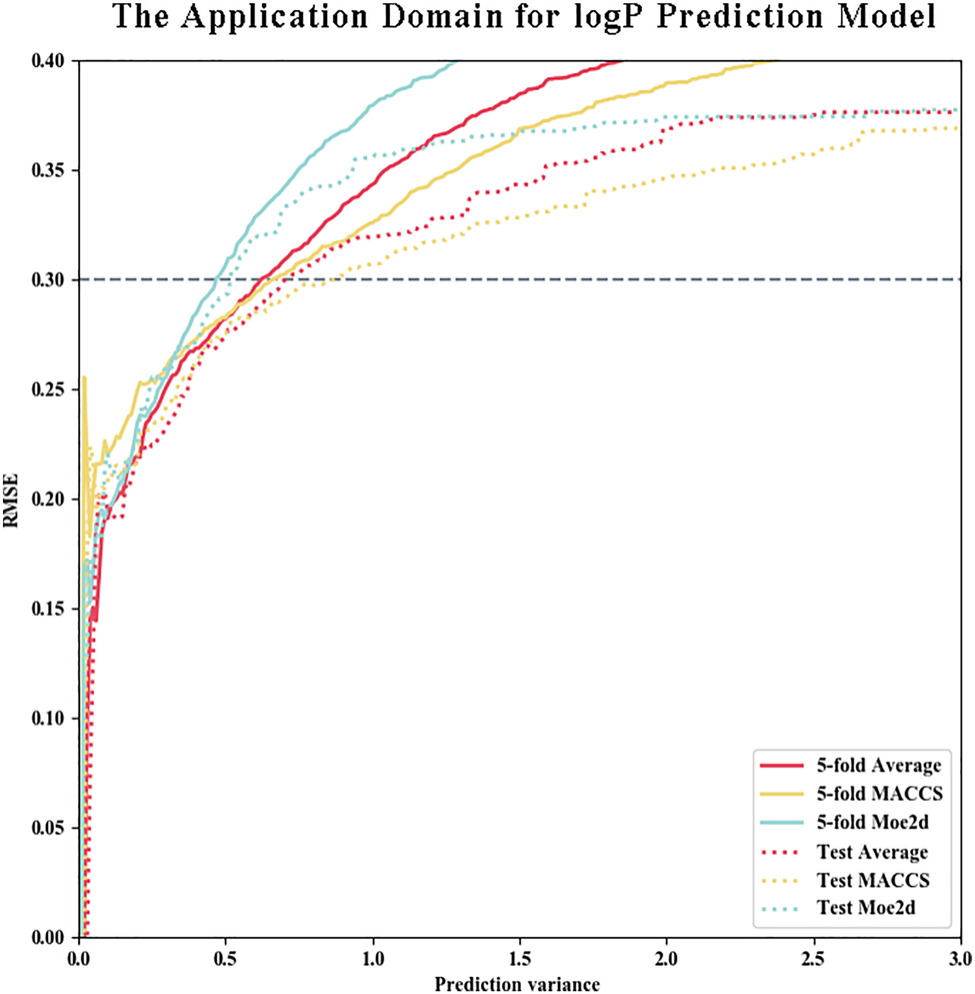
The application domain for LogP prediction model. The x-axis represents the standard derivation of the predicted data and the y-axis represents the value of RMSE, which is calculated by stepwise adding molecules with large prediction variance. Above figure has indicated that with the increase of variance threshold, the accuracy of predicted data will improve

After constructing the reliable models, the next step is to determine the appropriate AD threshold for the detection of accurately predicted molecules. To achieve it, we collected the standard deviation of the fivefold cross-validation and test set prediction results and then calculated the RMSEs of the molecules separately by the stepwise addition of molecules with larger prediction variance values (Fig. 2).

It can be seen from Fig. 2 that with the stepwise addition of molecules with large prediction variance, the prediction performance defined by the RMSE continuously deteriorated, suggesting that the prediction accuracy of each molecule could indeed be reflected by the prediction variance values to some extent, which indicated the utility and credibility of the AD method applied in this pipeline. Finally, taking RMSE = 0.300 as a threshold, we chose the compounds with an average prediction variance of less than 0.6 as the accurate prediction results. After the removal of the duplicates, 16,821 molecules from the ChemBridge and ChemDiv databases were compiled for the subsequent MMP calculation, and they substantially expanded the data size for further MMPA.

For the MMP calculation, both the experimental and accurately predicted data used the configuration of cutting all acyclic single bonds and a maximum of three cuts. Only the transformations containing more than 10 pairs and passing the Wilcoxon signed rank test (alpha = 0.05) were regarded as the final rules (see Additional file 1). For the experimental dataset, 1,367,650 MMPs and 10,650 unique transformations with more than 10 groups were generated, of which 8728 rules were obtained. During the detection of experimental rules, the standard deviation (SD) and standard error of mean (SEM) of different rules were calculated and summarized. As shown in Additional file 4: Fig. S1, with the increase of chemical clusters (calculated by the combination of Morgan fingerprint and a Tanimoto cut-off of 0.7), the SD of rules mean value first increased, but after a point, it became stable and almost unchanged. For the value of SEM, it firstly increased and then became stable, which is followed by a decrease finally. Such results have shown that transformation rules with more MMPs and chemical clusters are more likely to be generalized and credible, which have also indicated the importance of data expansion. For the accurately predicted dataset, 75,872 rules were finally obtained. After the combination of experimental and predicted data, the numbers of the unique transformations with more than 10 groups (104,336) and rules (91,510) were approximately ten times larger than those obtained from the experimental dataset, illustrating that the scale of MMPs can be significantly expanded by the application of the MMPA-by-QSAR pipeline.

**Fig. 3:**
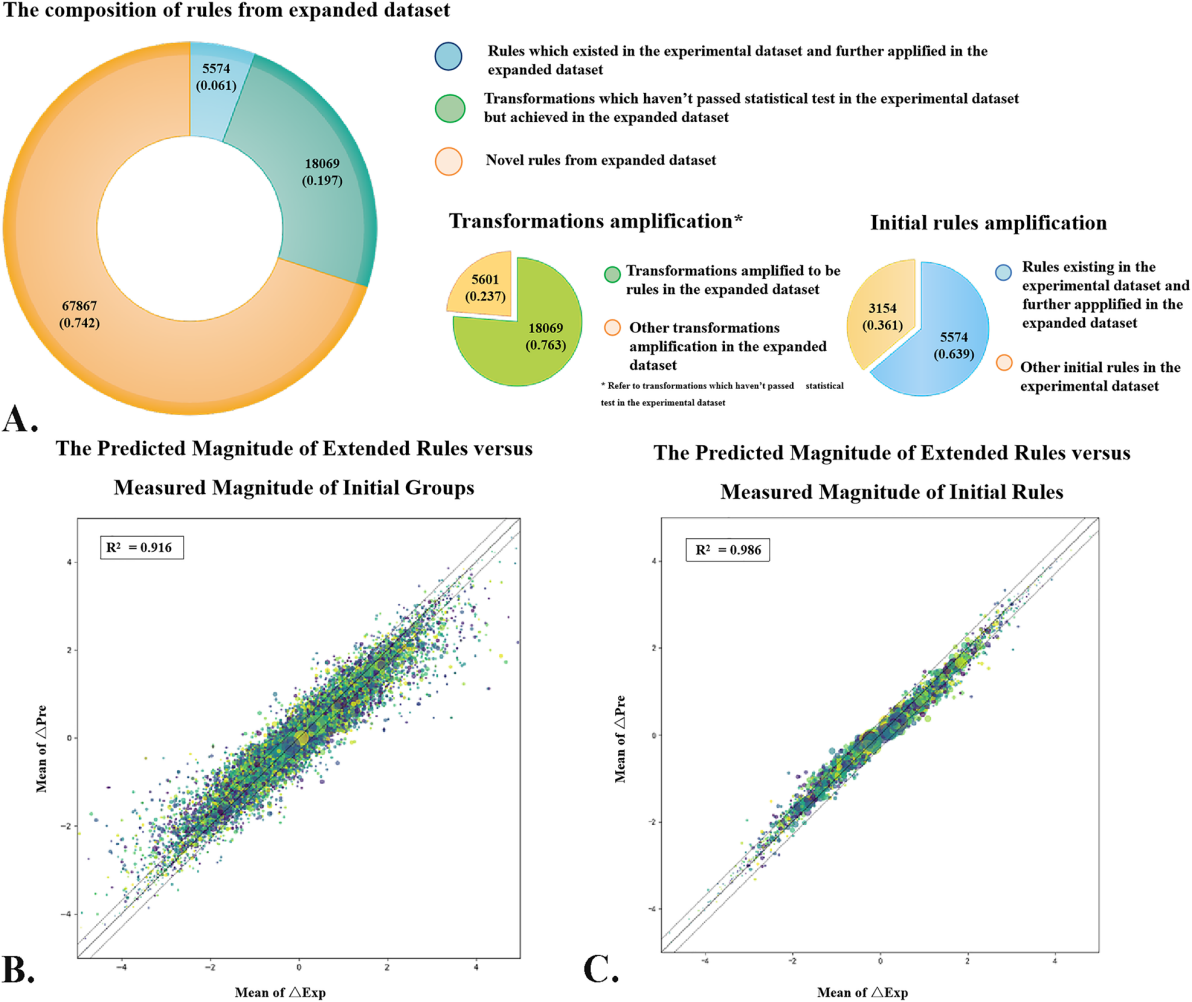
The relationship between the rules from expanded dataset and experimental dataset. **A** The composition and amplification effect of rules and transformations by expanded dataset. **B** The plots of the extended rules versus the measured magnitude of the property change for experimental transformations. **C** The plots of the extended rules versus the measured magnitude of the property change for experimental rules. Above figures have that MMPA-by-QSAR method can be used for transformation and rules amplification, where the expanded rules also agree well with the magnitude and directionality of the experimental rules

In addition to the expansion of the whole rules, MMPA-by-QSAR enables the amplification of existing ones by providing more evidence of the observed effect. As shown in Fig. 3A, a total of 23,680 transformations which haven’t passed the statistical test in the experimental dataset analysis have been amplified, of which 76.3% (18,069) were converted into rules in the expanded dataset analysis. For existing rules, 5574 rules from the experimental dataset analysis have also been amplified according to the increase of both MMPs and chemical clusters. To demonstrate the reliability of the MMP rules generated by the MMPA-by-QSAR pipeline, we continued to compare the magnitude and directionality of the rules from the expanded dataset with those from the experimental set. As shown in Fig. 3B, C, the rules derived from the expanded dataset well agreed with those derived from the experimental dataset with an R^2^ of 0.986, while the agreement between the expanded rules and experimental transformations which did not pass the statistical test was slightly lower with an R^2^ of 0.916. These results are not surprising because the number limitation of the initial groups can also negatively affect the accuracy of property change estimation.

To further demonstrate the credibility of the rules proposed by prediction models, we applied the newly predicted rules (which have never been derived from the experimental data) to the experimental data for comparison (Table 3). Clearly, our predicted rules are in qualitative agreement with these actual experimental data. The above results have indicated that to some extent, the MMPs calculated from MMPA-by-QSAR pipeline are useful for rules expansion and amplification, which is valuable for lead optimization. In general, more design principles for lead optimization and modification can be extracted through the application of this MMPA-by-QSAR pipeline by combining experimental and accurately predicted data, which greatly increases the scope and efficiency of MMPA.

**Table 3:**
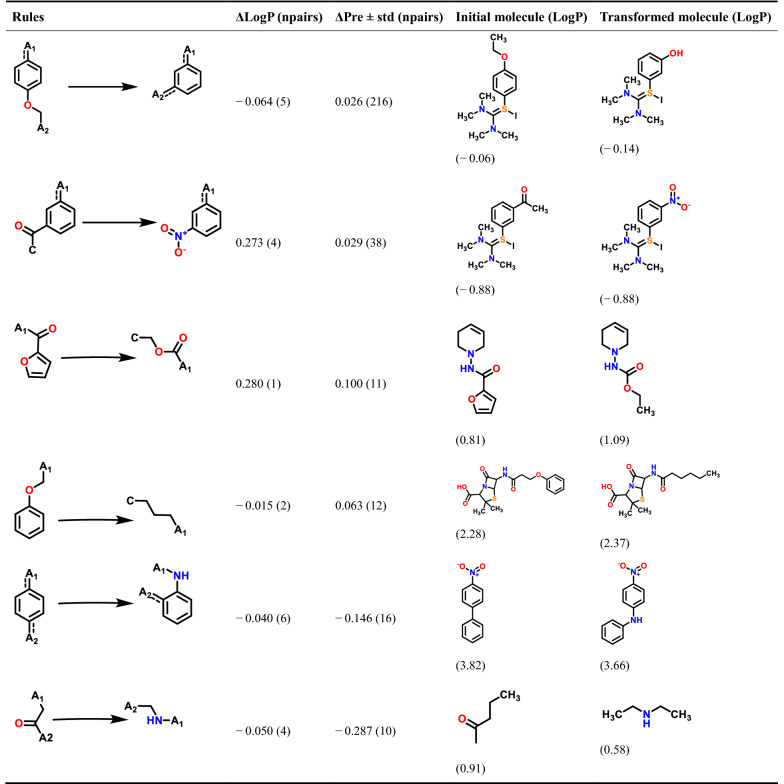
The application of the novel transformations from predicted data to experimental data

### MMPA of hERG dataset

The human ether-à-go-go-related gene (hERG) codes a protein known as Kv11.1, the α-subunit of a potassium-ion channel that mediates the inward repolarizing current as a part of the voltage cycle displayed in the electrocardiogram and is closely related to cardiotoxicity. Several drugs, such as terfenadine, astemizole, and cisapride, have been withdrawn because of their ability to inhibit hERG and thus induce QT-interval prolongation [48]. Therefore, the early-phase optimization of potential hERG inhibitors is highly important. To construct reliable hERG chemical transformation rules, 13,384 compounds containing 6736 hERG blockers (IC50 ≤ 10 μM or ≥ 50% inhibition at 10 μM) and 6,648 non-inhibitors (IC50 > 10 μM or < 50% inhibition at 10 μM) were collected from the ADMETlab webserver [49, 50]. The MACCS and MOE2D descriptors and four ML algorithms were used to construct the hERG prediction models, and the consensus model was also established to further improve the prediction performance. The compounds through the molecular preparation process were partitioned into the training set (4321 hERG inhibitors and 4254 non-inhibitors), the validation set (1078 hERG inhibitors and 1063 non-inhibitors), and the test set (1346 hERG inhibitors and 1331 non-inhibitors), which were used for model construction, hyper-parameter optimization, and model evaluation, respectively. The statistical results of the 10 fivefold cross-validation and test set predictions are summarized in Table 4.

**Table 4:** Performance of the hERG prediction models derived from different combinations of ML algorithms and descriptor sets

	Fivefold cross-validation				Test set			
	SE	SP	ACC	AUC	SE	SP	ACC	AUC
MACCS								
GB	0.873	0.84	0.857	0.925	0.883	0.860	0.871	0.939
RF	0.857	0.803	0.830	0.902	0.871	0.809	0.840	0.915
SVM	0.836	0.851	0.843	0.914	0.846	0.861	0.854	0.929
XGBoost	0.874	0.84	0.857	0.926	0.880	0.863	0.872	0.939
MOE2D								
GB	0.878	0.855	0.866	0.934	0.896	0.865	0.880	0.942
RF	0.872	0.844	0.858	0.925	0.883	0.839	0.861	0.935
SVM	0.883	0.856	0.869	0.936	0.854	0.841	0.848	0.920
XGBoost	0.883	0.856	0.869	0.936	0.887	0.866	0.877	0.945
Consensus model	0.865	0.882	0.874	0.935	0.894	0.865	0.879	0.946

**Fig. 4:**
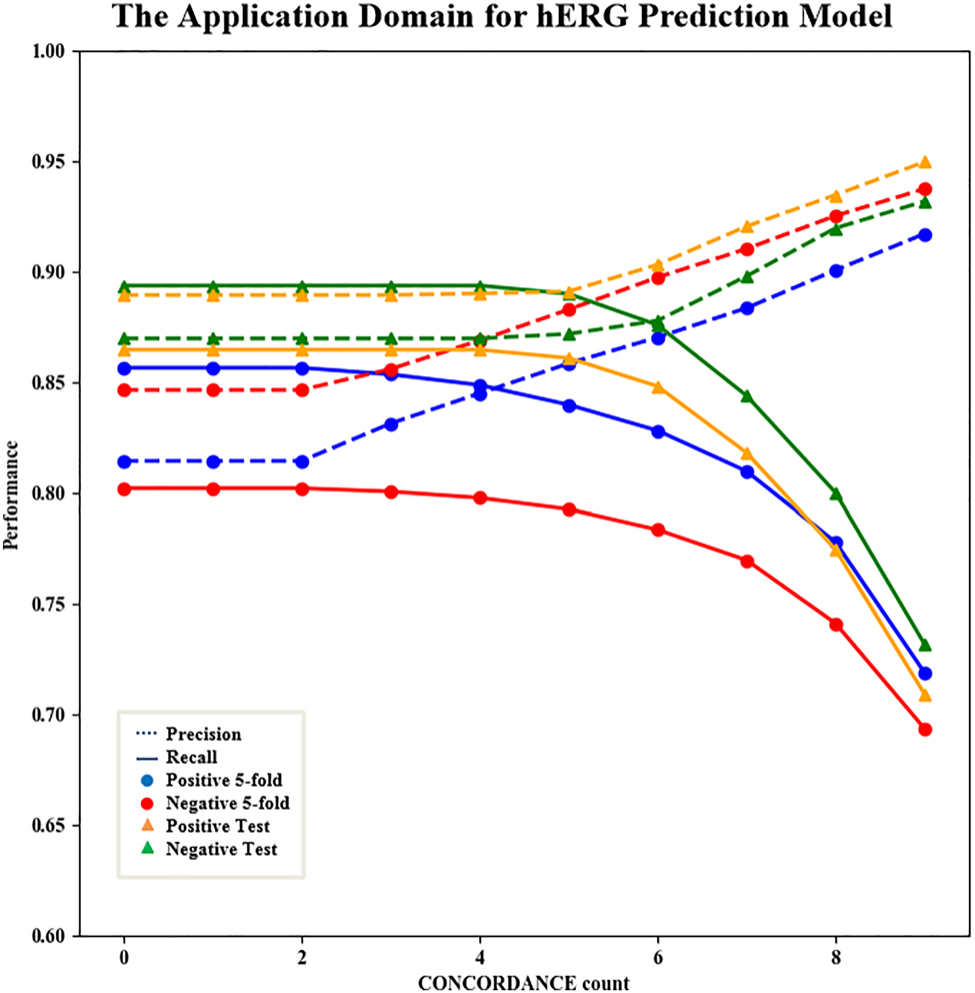
The AD evaluation of the hERG prediction model. The x-axis represents the amount of CONCORDANCE and the y-axis represents the value of precision and recall. Above figure has shown that with the increase of CONCORDANCE, the value of precision will increase and recall will decrease

Table 4 reveals that most prediction models performed well for both the training and test sets, with an average accuracy of 0.858 and an average AUC of 0.925 for the fivefold cross-validation, and an average accuracy of 0.864 and an average AUC of 0.934 for the test set. As the prediction performance of the consensus model (accuracy = 0.874/0.879 and AUC = 0.935/0.946 for the fivefold cross-validation/test set) is superior to those of the individual models, the consensus model is used as the final hERG prediction model. Similarly, to detect the appropriate AD threshold, we summarized the CONCORDANCE values of the fivefold cross-validation and test set predictions, and recorded the changes of the values of precision and recall with stepwise addition of molecules with larger CONCORDANCE counts (Fig. 4).

Figure 4 shows that with increasing the CONCORDANCE count, the precision value initially remains stable and then increases, indicating that the prediction results agree upon by most models are more reliable than those with low approval. It is also not surprising to see that the recall value slightly decreases with the increase of the CONCORDANCE count, as the high limitation implies strict screening and possible loss. The above results have indicated the utility of the AD evaluation of this pipeline for the classification task, which also enhances the credibility for further MMP calculation and analysis. Taking the full CONCORDANCE score as the final limitation, the potential hERG inhibitors predicted with a probability of more than 0.95 and non-inhibitors predicted with a probability of less than 0.025 were compiled for data expansion. After such harsh selection, 40,700 non-inhibitors and 48,717 potential inhibitors were combined with the experimental molecules as the expanded dataset. MMPA was performed based on both the experimental and expanded datasets with a configuration of cutting all acyclic single bonds and a maximum of three cuts. To ensure the reliability and utility, the final transformation rules need to satisfy the following requirements: number of pairs exceeds six, potential hERG inhibition possibility decreases, and a binomial distribution test for discrete variables is passed (probability of success = 0.5, alpha = 0.05). The results are summarized in Additional file 2.

Compared with the MMPA based on continuous data, the analysis of labeled data is much more difficult and narrowed. This is also the reason why the comprehensive MMPA-by-QSAR pipeline is urgently needed for chemical transformation mining. For the experimental dataset, only 9983 chemical transformations and 99 transformations with more than six groups were identified, of which only three chemical rules passed the statistical significance test. Similar to the logP research, more chemical transformations have been amplified through the process of the MMPA-by-QSAR pipeline. For the expanded dataset, 45 chemical rules were generated, of which three chemical rules and two transformation conversions from the experimental dataset were included.

To explore the credibility of the newly added chemical rules from the expanded dataset, we compared them with the data reported by previous hERG studies. As shown in Table 5, most toxic substructures contain the piperidine or piperazine moieties with positively charged nitrogen atoms. According to previous studies, it has been widely accepted that a positively charged nitrogen generally increases the likelihood of hERG binding due to cation–π interactions with Tyr652. Tertiary amine groups linked by a hydrophobic tail are also potential hERG-binding fragments, as the hydrophobic part of such fragments may engage in strong van der Waals or hydrophobic interactions with hERG residues such as Phe656. To avoid potential hERG inhabitation, several transformation directions have been proposed in the expanded rules, such as the substitution of carbonyl linker and methylene linker. The above analysis not only shows the utility of MMPA but also indicates the advantages of the MMPA-by-QSAR pipeline in the chemical transformation mining of small experimental datasets. It can achieve both the amplification of existing rules and the enrichment of newly credible transformations. By rational application of this pipeline, it is believed more structural optimization guidance can be acknowledged and applied for the promotion of drug design and research.

**Table 5:** The comparison of the hERG rules from experimental data and expanded data

Rules	Experimental data		Expanded data	
	Toxicity change^a^	npairs	Toxicity change^a^	npairs
	− 0.026	344	0	22,024
	− 0.046	322	− 0.001	13,353
	− 0.040	151	− 0.002	3657
	None	None	− 1	9
	None	None	− 1	15
	None	None	− 1	6
	None	None	− 1	8
	None	None	− 1	6

^a^Toxicity change = (the number of pairs that increase toxicity − the number of pairs that decrease toxicity)/the number of all pairs

## Conclusion

Drug discovery has always been hindered by the problem of lead compound optimization. MMPA, a useful tool for efficiently extracting and summarizing the relationships between structural transformation and property change, is suitable for local structural optimization tasks. In particular, the integration of MMPA with QSAR modeling can further strengthen the utility of the former in molecular optimization navigation, especially for small experimental datasets. Herein, an integral and semi-automated procedure was constructed for MMPA and MMPA-by-QSAR construction and application, including molecule preparation, QSAR model construction, applicability domain evaluation, and MMP calculation and application. Easy-management and the integration of QSAR and MMPA of this workflow allow medical chemists for wider and deeper chemical transformation mining of experimental datasets, in which molecule preparation and AD limitation ensure the consistency and credibility of experimental and predicted data, respectively. The systematic statistical test and MMP context clustering have further guaranteed the efficacy and generality of the summarized rules in practical molecular optimization. Furthermore, the application of negative design screening tool benefits the quality of optimized molecules by filtering out molecules with undesirable properties or substructures. To demonstrate the utility of this pipeline, two examples covering regression and classification tasks were provided to better understand the utility of this pipeline and demonstrate the efficiency of comprehensive MMP-based analysis and the reliability of the MMPA-by-QSAR method (Additional files 5, 6). The rational application of this pipeline should allow chemists to draw more useful information on chemical transformations and appropriate optimization navigation across limited datasets, thus increasing the efficiency and success rate for the development of productive activities.

## Supplementary Information

**Additional file 1.** The statistical results of MMPA for the experimental and expanded LogP dataset.

**Additional file 2.** The statistical results of MMPA for the experimental and expanded hERG dataset.

**Additional file 3.** The user tutorial of the MMPA and MMPA-by-QSAR pipeline.

**Additional file 4: Figure S1.** The comparison of (A) SD and (B) SEM of rules with different chemical clusters. **Table S1.** The introduction of the computational tools.

**Additional file 5.** The MMPA and MMPA-by-QSAR pipeline for classification task.

**Additional file 6.** The MMPA and MMPA-by-QSAR pipeline for regression task.

## Data Availability

The KNIME workflows for both labeled and consecutive data are freely and the datasets supporting the conclusions of this article are included within the article.

## Abbreviations

ADME: Absorption, distribution, metabolism and elimination
ML: Machine learning
ML: Deep learning
MMP: Molecular matched pair
MMPA: Matched molecular pair analysis
RF: Random forest
XGBoost: Extreme gradient boosting
SVM: Support vector machine
GB: Gradient boosting
Q2/R2: Squared correlation coefficient
RMSE: Root mean squared error
MAE: Mean absolute error
TP: True positive
TN: True negative
FP: False positive
FN: False negative
ACC: Accuracy
SE: Sensitivity
SP: Specificity
F1: Index F
AD: Applicability domain
TREE_SD: Variation of prediction among random forest trees
AD: Applicability domain
hERG: Human ether-à-go-go-related gene
