# Supplementary material for: Semi-automated workflow for molecular pair analysis and QSAR-assisted transformation space expansion
Source: J Cheminform. 2021 Nov 13;13:86. doi: 10.1186/s13321-021-00564-6 (PMC8590336; doi:10.1186/s13321-021-00564-6)
Supplement: Supplementary file 3 — Additional file 3. The user tutorial of the MMPA and MMPA-by-QSAR pipeline. [file 13321_2021_564_MOESM3_ESM.pdf]

User Guide for MMPA-by-QSAR KNIME pipeline

CBDD Group (Computational Biology & Drug Design Group),
Xiangya School of Pharmaceutical Sciences, Central South University

CONTENT

USER GUIDE 0

BRIEF INTRODUCTION 2

COMPUTATIONAL TOOL 3

MODULE INTRODUCTION 4

MOLECULE PREPARATION 4

Molecule inspection and salt deletion 4

Structure validation 5

Normalization and duplicate deletion 5

MODEL CONSTRUCTION AND EVALUATION 6

Descriptor calculation 7

Feature selection 8

Algorithm selection 8

Parameter optimization 9

5-fold cross validation, test set and external set evaluation 11

Applicability domain 12

External Dataset prediction 13

MMP CONSTRUCTION AND APPLICATION 14

Fragment calculation 14

Statistical test 16

MMP application and further evaluation 16

Brief introduction

The discovery of drug candidates which can block or activate the target protein of interest involves extensive virtual and experimental screening, which also counts about 30–40% of the total time for drug development. During the searching of drug candidates, it is hard to directly find an optimal drug candidate with adequate therapeutic potency, appropriate ADME property, selectivity, and reassuring safety. Therefore, the efficiency of lead optimization will produce a significant influence on the process of new drug research and development. MMPA, a useful tool to efficiently extract and summarize the relationship between chemical structure transformation and property change, is suitable for the local structural optimization task. Especially, the integration of MMPA with QSAR modeling can further strengthen the utility of MMPA in the molecular optimization navigation. In this research, a new semi-automated procedure on KNIME was constructed to support scientists for MMPA and QSAR-assisted-MMPA construction and application, including molecule preparation, QSAR model construction, applicability domain evaluation, MMP construction and application. This pipeline is constructed by CBDD Group (Computational Biology & Drug Design Group), Xiangya School of Pharmaceutical Sciences, Central South University.

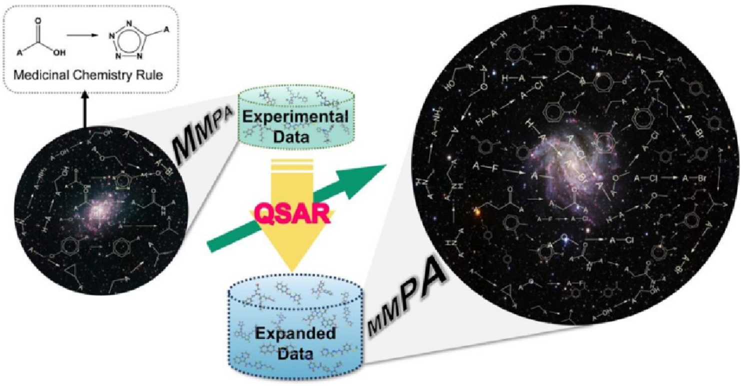

Figure 1. The concept of QSAR-assisted-MMPA

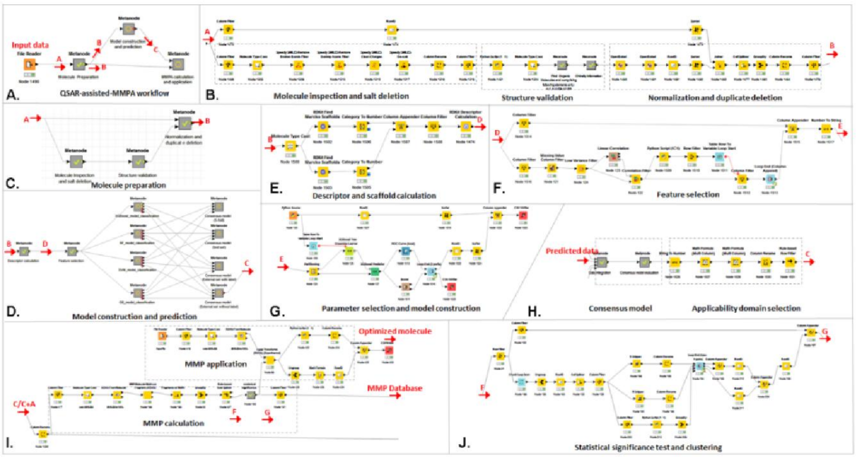

Figure 2. The outline of the QSAR-assisted-MMPA pipeline

Computational tool

- **KNIME:**
  The edition of KNIME Analytics Platform should be higher than 4.1.2. The related extensions will be installed after the import of the KNIME automatically. Before calculation, the Python environment and R path should be settled correctly, which can be seen in the "File>>>Preferences>>>KNIME>>>Python" and "File>>>Preferences>>>KNIME>>>R", respectively. More information about KNIME is provided in https://www.KNIME.com/.
- **R:**
  The R version should be higher than 3.6.0.
- **Python:**
  The python version is Python 3.
  Dependencies: rdkit, pandas, sklearn, numpy, matplotlib and Scopy (https://github.com/kotori-y/Scopy).

Table 1. The introduction of the computational tools

Name	Description
RDKit	Scaffold calculation, Descriptor/Fingerprint calculation and MMP chemical diversity calculation.
pandas	Python-data analysis
sklearn	Model construction
numpy	Python-n-dimensional arrays
matplotlib	Figure visualization
Scopy	Molecular pretreat and Descriptor/Fingerprint calculation
OpenBabel	Canonical SMILES and InCHIkey calculation

Module introduction

The discovery of drug candidates which can block or activate the target protein of interest involves extensive virtual and experimental screening, which also counts about 30–40% of the total time for drug development. During the searching of drug candidates, it is hard to directly find an optimal drug candidate with adequate therapeutic potency, appropriate ADME property, selectivity, and reassuring safety. Therefore, the efficiency of lead optimization will produce a significant influence on the process of new drug research and development. MMPA, a useful tool to efficiently extract and summarize the relationship between chemical structure transformation and property change, is suitable for the local structural optimization task. Especially, the integration of MMPA with QSAR modeling can further strengthen the utility of MMPA in the molecular optimization navigation. In this research, a new semi-automated procedure on KNIME was constructed to support scientists for MMPA and QSAR-assisted-MMPA construction and application, including **molecule preparation**, **QSAR model construction and evaluation**, and **MMP construction and application**.

Molecule preparation

Check and preparation of molecular structures are the necessary prerequisites for structural analysis. For QSAR model derivation, the quality of data is extremely important as it strongly affects the final robustness and predictive power of the model. For MMPA, the compounds used for analysis must be in a consistent salt, charge and tautomeric state. Considering the requests from QSAR analysis and MMPA, the molecule preparation module is designed with three parts: molecule inspection and salt deletion, structure validation and normalization and duplicate deletion.

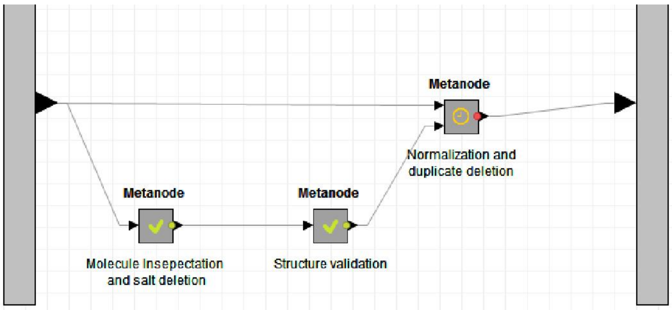

Figure 3. The constitute of Molecule Preparation module

Molecule inspection and salt deletion

Only the column of Smiles will be selected in the following molecular preparation process. In the molecule inspection and salt deletion part, the input molecules with broken bonds (i.e bonds where a numeric bond start index has no corresponding end index) or dummy atoms (i.e. *, [*], [1*] etc) will be filtered out. In addition, molecular charge style will be unified and the multiple components will be manipulated, where only the components with the most number of heavy atoms are kept.

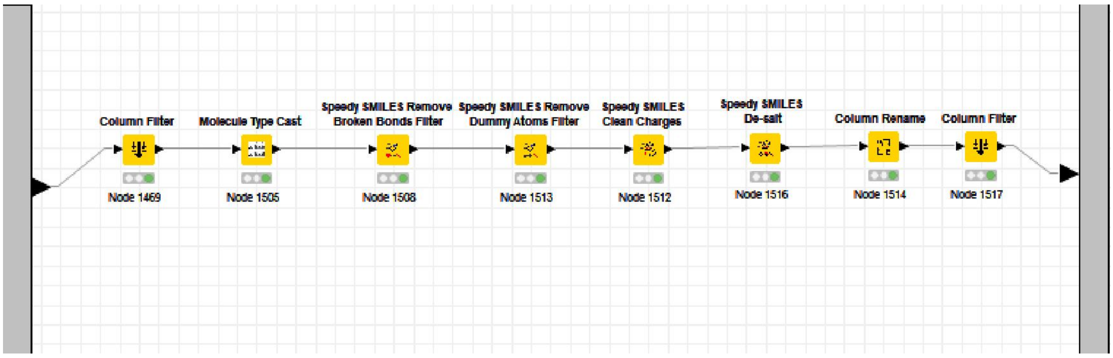

Figure 4. The constitute of Molecule inspection and salt deletion

Structure validation

In the structure validation part, molecules will firstly be prepared by the ScoPretreat module of Scopy library, which involves normalization of functional groups to a consistent format, tautomer enumeration, generation of fragment, isotope, charge, tautomer or stereochemistry insensitive parent structures and validations to identify molecules with unusual and potentially troublesome characteristics. To ensure the prediction accuracy and efficiency, only compounds without uncommon atoms are used for QSAR model construction and prediction. Therefore, compounds with uncommon elements except (H, C, N, O, F, Br, I, Cl, P and S) will be tagged. In addition, this pipeline mainly focuses on the 2D QSAR model construction and MMPA. If standard descriptors are calculated from 2D representation of chemical structure, then any pair of enantiomers or disstereoisomers will be formally recognized as duplicates. Therefore, the molecules with chirality information will also be tagged.

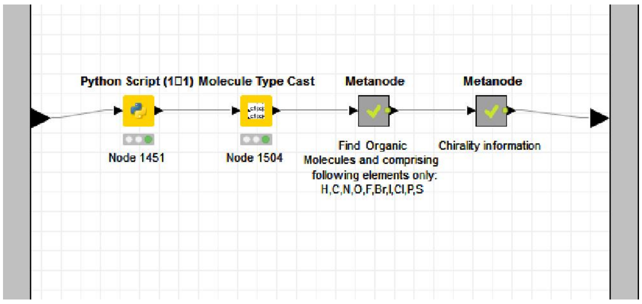

Figure 5. The constitute of Structure validation

Normalization and duplicate deletion

In the normalization and duplicate deletion part, Canonical SMILES and the corresponding InChIKey will be calculated. Latter the processed Smiles information will be combined with other input information. According to the InChIKey information, the duplicate can be deleted latter.

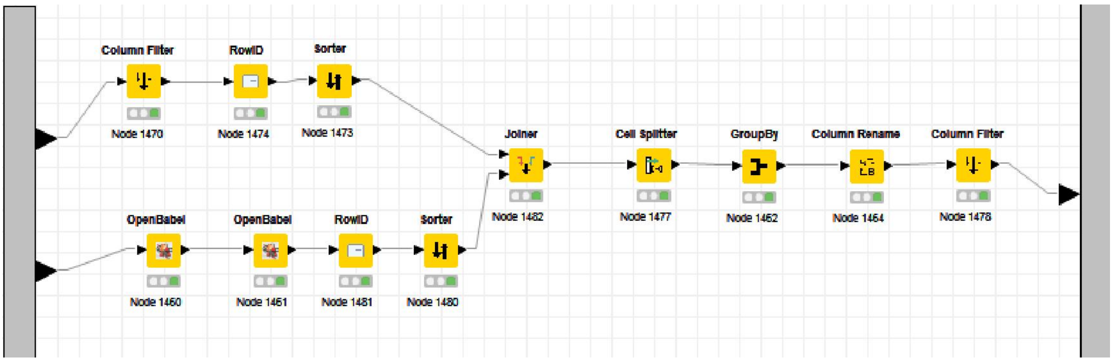

Figure 6. The constitute of Normalization and duplicate deletion

Note:

1. The format and related information of the input and output data.

Table 2. The format information the input and output table

File	Column name	Related information
Input file	Smiles	Smiles information of input molecule
	Value	Aim property (for consecutive property)
	Label	Aim property (for binomial property)
Output file	InChIKey	The calculated InChIKey information of the Final Smiles
	Warning (elements)	Tag for compounds with uncommon atom (except H, C, N, O, F, Br, I, Cl, P and S)
	Warning (inorganic)	Tag for inorganic molecule
	Warning (stereochemistry)	Tag for compounds with stereochemistry information
	Initial Smiles	Smiles information of input molecule
	Final Smiles	Smiles information of processed molecule
	Mean (Value)/Mean (Label)	The mean value of the aim property
	Concatenate* (Value)/Concatenate* (Label)	The collection of possible values of the aim property, which can be used as the reference for following data process

2. Manual examination

It should be noted that though the curation of data can be achieved automatically, it is recommended to process manual examination of the final processed molecules to ensure the model predictivity and MMPA credibility.

Model construction and evaluation

The construction of a high-accuracy and credible prediction models is of utmost important in the process of QSAR-assisted-MMPA. In the part of model construction and evaluation, descriptors calculation and selection, model parameter optimization and construction, applicability domain and model performance evaluation are

included to provide the comprehensive evaluation of the model performance and the accurate prediction results.

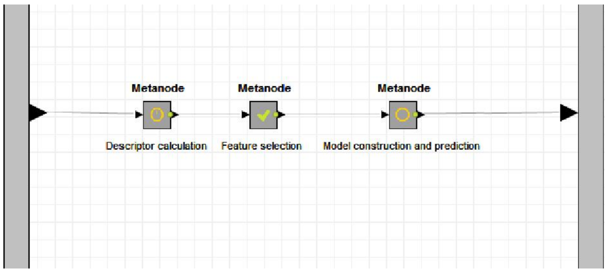

Figure 7. The constitute of Model construction and evaluation module

Descriptor calculation

In the descriptors calculation part, QSAR-assisted-MMPA pipeline provides 17 frequently used fingerprints and descriptors, including Moe2d descriptor, RDKit descriptor, Morgan fingerprint, FeatMorgan fingerprint, AtomPair fingerprint, Torsion fingerprint, Avalon fingerprint, Layered fingerprint, MACCS fingerprint, EFG descriptors, PubChem descriptors, Estate descriptors, GhoseCrippen descriptors and IFG descriptors. These fingerprints and descriptors can provide both structure and physicochemical property information, thus providing good performance in model construction. In addition, the calculation of two molecular scaffold types Murcko scaffolds and carbon skeletons are also provided in this pipeline for the chemical diversity exploration of datasets.

Table 3. The summary of descriptors, fingerprints and scaffolds in QSAR-assisted-MMPA pipeline

Section	Features	Number of descriptors/fingerprints
RDKit Descriptor Calculation	RDKit descriptor	119
Moe Descriptors*	Moe2d descriptor	600
	Morgan (bit and count)	1024
	FeatMorgan (bit and count)	1024
	AtomPair (bit and count)	1024
RDKit Fingerprint/ RDkit Count-Based Fingerprint	Torsion (bit and count)	1024
	RDKit (only bit)	1024
	Avalon (only bit)	1024
	Layered (only bit)	1024
	MACCS (only bit)	167
	MACCS descriptors	167
	EFG descriptors	583
	PubChem descriptors	881
Scopy_Scorepresent module	Estate descriptors	79
	GhoseCrippen descriptors	110
	IFG descriptors	undefined
	Daylight fingerprints	2048
Scaffold calculation	Murcko scaffolds	Removing all substituent groups but conserving the linkers between ring

		systems
	Carbon skeletons	Changing all heteroatoms of Murcko scaffold into carbon atoms and all bond orders into single bond

*:The calculation of Moe2d descriptors needs license.

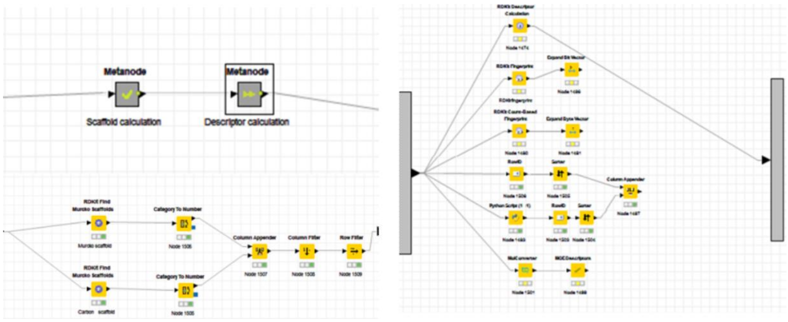

Figure 8. The constitute of Descriptors calculation part

Feature selection

The aim of feature selection part is to remove irrelevant variables and consequently improve the reliability and efficiency of prediction models. The feature selection is conducted by the following steps: (1) the descriptors whose variance were 0 or approached 0 were deleted; (2) if the correlation coefficient between two descriptors was higher than 0.95, only one was selected; (3) Recursive feature elimination algorithm for variable selection, take AUC/R2 as the evaluation index. Users can also adjust related limitation value according to their need.

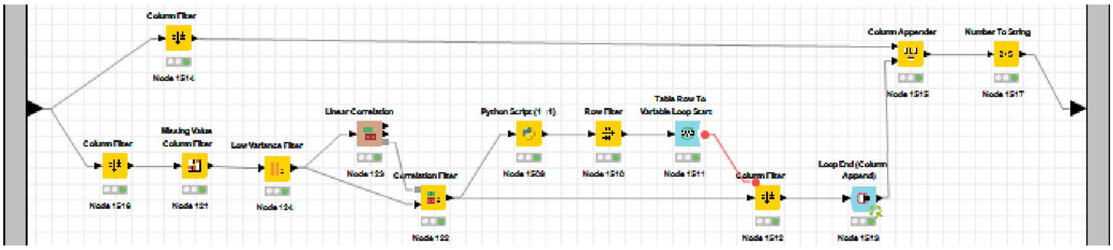

Figure 9. The constitute of Feature selection part

Algorithm selection

In the algorithm selection part, four machine learning algorithms are provided for the model construction: Random Forest (RF), XGBoost, SVM and Gradient Boosting (GB). According to the former experience, the construction of consensus model which draw prediction scores by averaging the outputs of multiple individual models is recommended to be applied as the final prediction result in this pipeline.

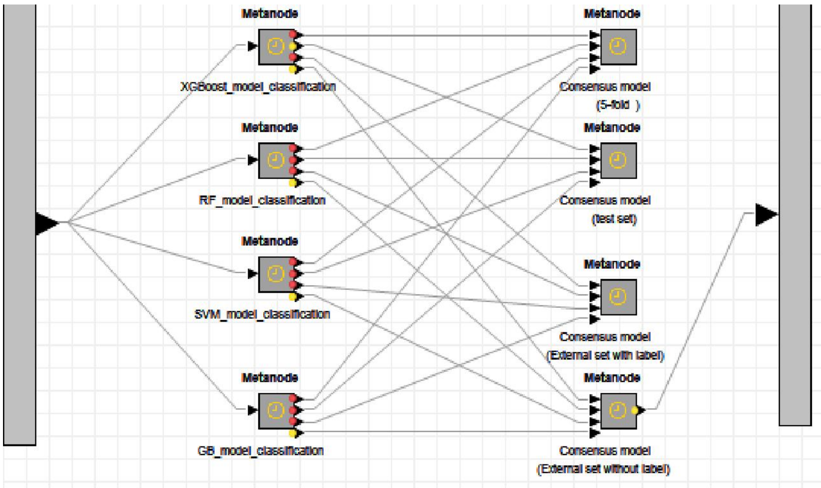

Figure 10. The constitute of Model construction and evaluation part (Take classification model as an example)

Parameter optimization

Considering the importance of parameters to model performance, QSAR-assisted-MMPA pipeline enables the grid search method to optimize the ability of prediction models. The overall prediction accuracy (ACC) and the squared correlation coefficient (R2) are used for classification and regression model evaluation, respectively. Following are important parameters of different algorithms and related recommended value range.

Table 4. The important parameters of different algorithms and related recommended value range

Algorithm	Parameter	Recommended range
Random Forest	Number of decision trees (n_tree)	From 500 to 2000, interval = 100
	Maximum fraction of features considered per split (max_features)	From 10 to 100, interval = 5
XGBoost	Learning rate (Eta)	From 0.01 to 0.3, interval = 0.02
	Maximum depth of a tree (maximum depth)	From 0.01 to 0.3, interval = 0.02
	Number of models to train in the boosting ensemble (boosting rounds)	From 500 to 3000, interval = 100
SVM	Penalty parameter (C)	From -5 to 5, interval = 0.5
	Gamma (gamma)	First selection: 0.01, 0.05 0.1, 0.5, 1, 5, 10
Gradient Boost	Number of decision trees (n_tree)	From 500 to 2000, interval = 100
	Maximum fraction of features considered per split (max_features)	From 10 to 100, interval = 5
	Learning rate (Eta)	From 0.01 to 0.3, interval = 0.02

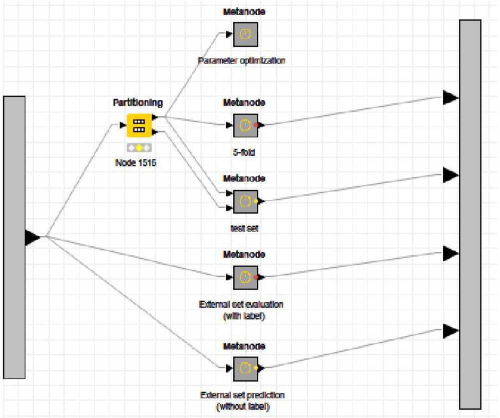

Figure 11. The constitute of Model construction (Take classification model as an example)

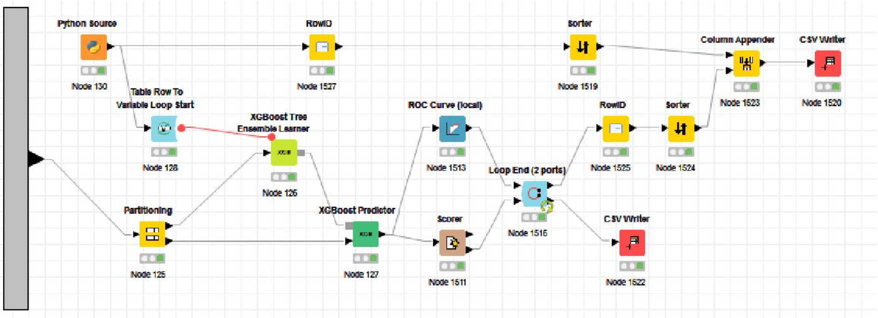

Figure 12. The constitute of Model parameter optimization

5-fold cross validation, test set and external set evaluation

To ensure that the prediction models have good generalization ability to the feature prediction, 5-fold cross validation, test set and external validation set are used for evaluation.

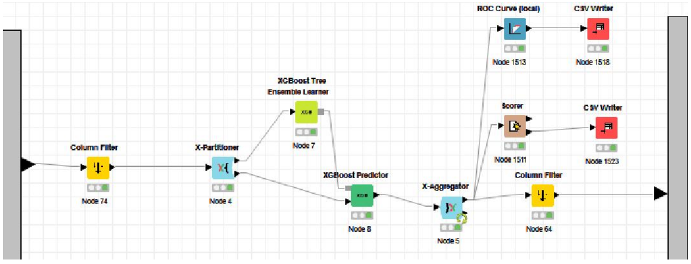

Figure 13. The constitute of 5-fold cross validation

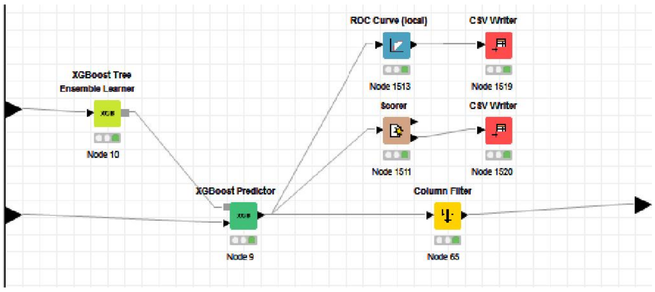

Figure 14. The constitute of test set evaluation

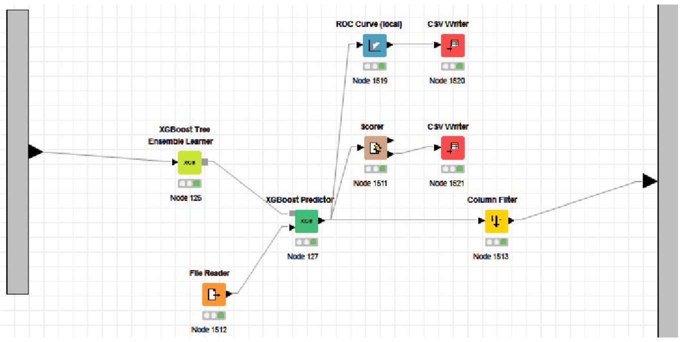

Figure 15. The constitute of external set evaluation

The following statistical parameters are used to evaluate the performance of prediction models: For classification models, true positives (TP), false negatives (FN), true negatives (TN), false positives (FP), the overall prediction accuracy (ACC), the prediction accuracy of the positive set (sensitivity, SE), the prediction accuracy of the negative set (specificity, SP), index F, precision, recall, the receiver operating characteristic (ROC) curve and the area under the receiver operating characteristic curve (AUC) are used to assess each model. For regression models, the squared correlation coefficient (Q2), the root mean squared error (RMSE), the mean absolute error of cross validation (MAE) are applied for the evaluation of model performance. These statistical parameters are defined as follows:

Table 5. The statistical parameters of model prediction performance

Category	Parameters	Definition	Meaning
Classification prediction models	True positive (TP)	Real Label = 1 and Predicted Label =1	Number of correctly classified positive results
	True negative (TN)	Real Label = 0 and Predicted Label =1	Number of correctly classified negative results
	False positive (FP)	Real Label = 0 and Predicted Label =0	Number of misclassified positive results
	False negative (FN)	Real Label = 1 and Predicted Label =0	Number of misclassified negative results
	Accuracy (Acc)	ACC = (TP+TN)/(TP+TN+FP+FN)	Overall prediction accuracy
	Sensitivity (SE)	SE = TP/(TP+FN)	Prediction accuracy of the positive set
	Specificity (SP)	SP = TN/(TN+FP)	Prediction accuracy of the negative set
	Precision	Precision= TP/ (TP+FP)	Efficiency of positive results prediction
	Recall	Recall= TP/ (TP+FN)	Coverage of positive results prediction
	Index F (F1)	F1 = 2Precison*Recall / (Precision + Recall)	Evaluation of the comprehensive performance of the models
	receiver operating characteristic (ROC) curve area under the Roc curve (AUC)	The probability that a randomly chosen positive example is ranked higher than a randomly chosen negative example	The performance of the classification model as its discrimination threshold is varied
Regression prediction model	Squared correlation coefficient (R2)	$R2 = 1 - \frac{\sum_{i=1}^{m}(y_i - \hat{y}_i)^2}{\sum_{i=1}^{m}(y_i - \bar{y}_i)^2}$	Squared correlation coefficient
	Mean absolute error of cross validation (MAE)	$MAE = \frac{1}{m}\sum_{i=1}^{m}(y_i - \hat{y}_i)^2$	Mean absolute error of cross validation
	Root mean squared error (RMSE)	$RMSE = \sqrt{(MAE)}$	Root mean squared error

Applicability domain

Any QSAR prediction of biological or physicochemical properties has limited value without an estimated

applicability domain (AD) of a model. Therefore, the estimation of AD of a model is necessary for the molecular prediction reliability. For classification models, CONCORDANCE method is used for AD detection, which reflects the number of models that give the same prediction that the current model does. For regression models, prediction variation errors calculated by RF models are used for the evaluation of model AD. To benefit the exploration of appropriate AD limitation, the graph of relationship between the AD limitation and model prediction performance will be depicted during the pipeline.

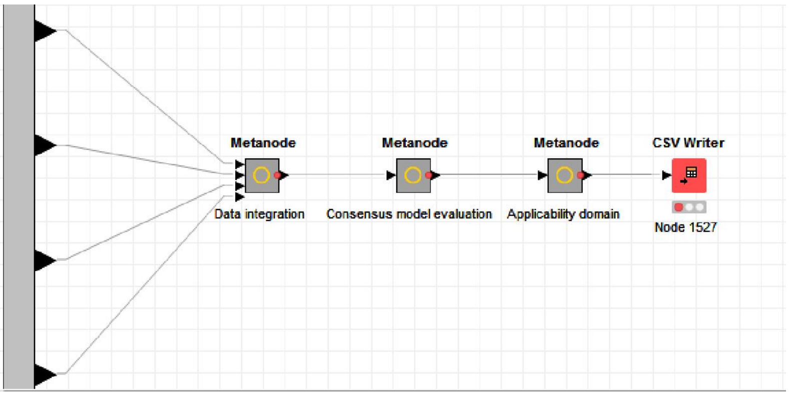

Figure 16. The constitute of consensus model and applicability domain

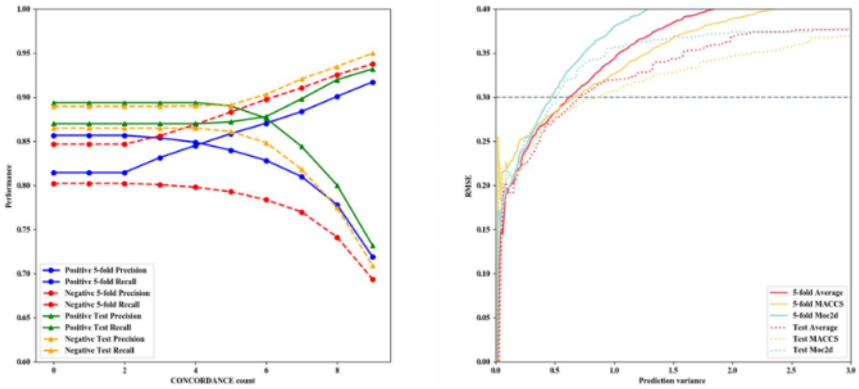

Figure 17. The example graphics of applicability domain exploration

External Dataset prediction

To better tackle the limited experimental data problem of matched molecular pair analysis (MMPA), MMPA-by-QSAR paradigm is proposed, for which QSAR modeling are firstly employed to make predictions for non-labeled data and subsequently MMPA are used for chemical transformation analysis based on the predicted compound activities. Therefore, the accurate predicted data can be used as the useful supplementary tool for more efficient and useful MMPA. Users can use the accurate-predicted molecules as the potential external dataset for MMPA.

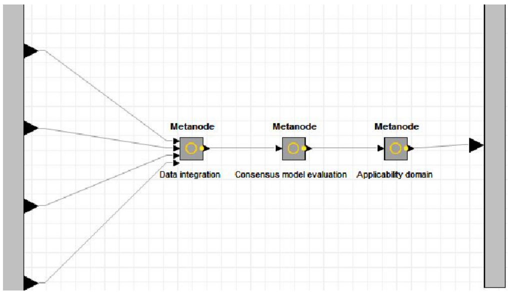

Figure 18. The constitute of accurate predicted molecules

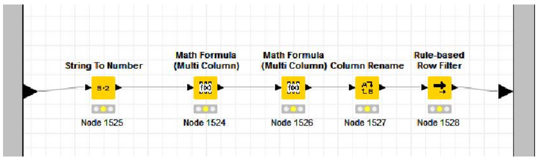

Figure 19. The constitute of accurate-predicted consensus model results selection

MMP construction and application

An MMP is generally defined as a pair of compounds that are interconverted by a well-defined chemical transformation, where the change between the pair is referred to the transformation and the invariant feature is referred to the context. Systematic extraction and summarization of MMPs from a large chemical database possess analytical and generative characters. Compared with the former optimization method, MMP owns both advantages of first-hand chemistry data and clear interpretation of the results. It summarizes direct information from chemical data with easy operation, which provides a wide range of functions, including suggesting what to make next, predicting the properties of a new compound, identifying cases where a structural change has a minimal effect on key properties (like bioisosteres), or simply to increase our understanding of the links between biology and chemistry. Concerning that MMPA focuses on the interesting local structural feature transformation rather than the whole molecule, which is more suitable for optimization problem. In the MMP construction and application part, it includes the calculation of MMP, statistical significance screening and further MMP application and optimized molecule evaluation.

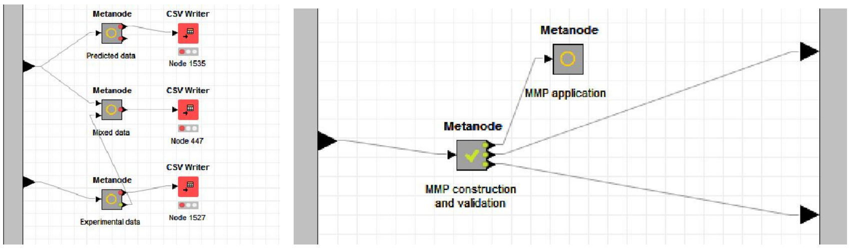

Figure 20. The constitute of MMP construction and application

Fragment calculation

In this pipeline, the MMPs were generated using an implementation of the Hussain and Rea algorithm, which identifies shared substructures by fragmenting each molecule and then storing and indexing all of the enumerated

fragments in an inverted-file-like structure. Following table has shown the parameters which can be adjusted during the fragment calculation process.

Table 6. The parameters of fragment calculation process

Part	Parameter	Option
Molecule and Fragmentation Options	Fragmentation type	1. "All acyclic single bonds"<br>2. "Only acyclic single bonds to rings"<br>3. "Only acyclic single bonds to either rings or to double bonds exocyclic to rings"<br>4. "Only single bonds to a heteroatom"<br>5. "Non-functional group single bonds"<br>6. "Matsy (One atom in ring, or a non-sp2 C atom bonded to a non-C atom)"<br>7. "Peptide Sidechains"<br>8. "Nucleic Acid Sidechains"<br>9. "User defined" (Using own (r)SMARTS fragmentation definition)
	Hydrogen manipulation	The addition of H before fragmentation and the deletion of H after fragmentation
Fragmentation Filtering Settings	Maximum number of cuts	The maximum number of cuts in a molecule (Range: 1~10)
	Complexity	The maximum number of fragments in a molecule possessing based on the number of possible fragmentable bond combinations
	Chirality	The manipulation of no undefined chiral centers
	Maximum number of heavy atoms of fragmentation	The maximum number of heavy atoms which are allowed to change between pairs
	Ratio of changing to unchanging part	The minimum ratio of changing to unchanging heavy atoms
Output Settings	The detailed information about processed fragments	1. The number of changing atoms<br>2. The ratio of constant / changing heavy atoms<br>3. The fragmentation failure reasons<br>4. Fragment rendering and appearance<br>5. Input column information
Fingerprints	The calculation and addition of graph distance fingerprint of the fragment "value"	Fingerprint length, radius, bond types and chirality information

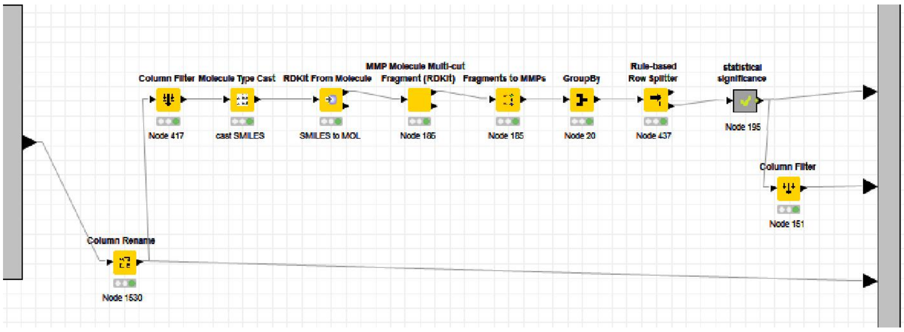

Figure 21. The constitute of fragment calculation

Statistical test

To ensure the credibility of compiled MMP, the minimum number of MMP pairs and statistical significance test are applied for the MMP screening. Wilcoxon Signed Rank Test for continuous variables (confidence interval = 0.95) and Binomial distribution test for discrete variables (probability of success = 0.5, confidence interval = 0.95) are used for statistical significance evaluation of continuous values and binomial values, respectively. Besides, to avoid false positive results, Benjamini Hochberg is used for multiple testing correction. Since the chemical transformation rules are generalized over many compound pairs, the context information of specific MMP is important for the detection of the specificity and generalizability of the structural change. Considering that, the fingerprint of the entire molecular context and corresponding clustering are provided in this pipeline.

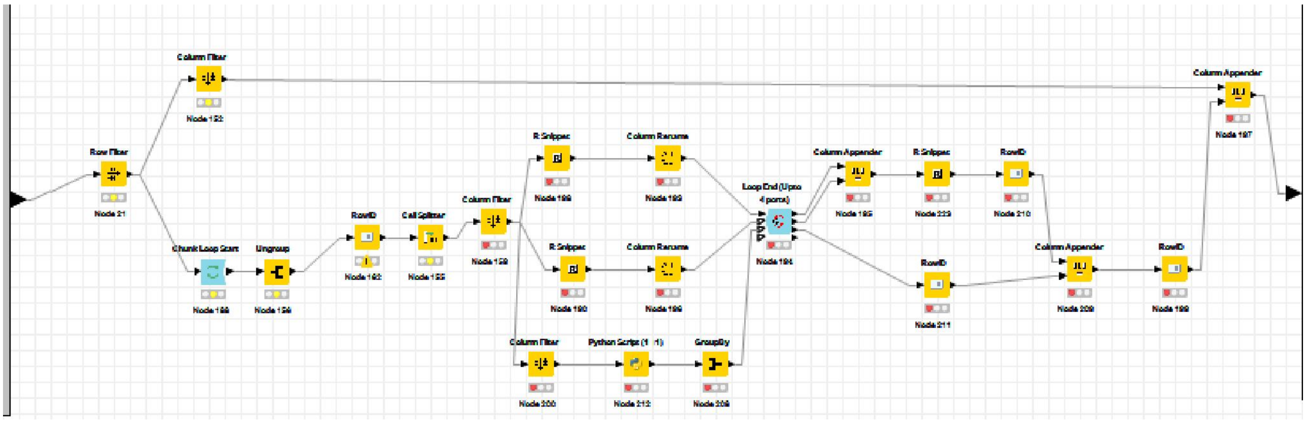

Figure 22. The constitute of statistical test (label data)

MMP application and further evaluation

The final MMPA chemical transformation rules can be applied to the aim property optimization of initial molecules, where the optimized molecules can be further evaluated by drug-likeness index and substructure rules based on the application of Scopy library (https://github.com/kotori-y/Scopy).

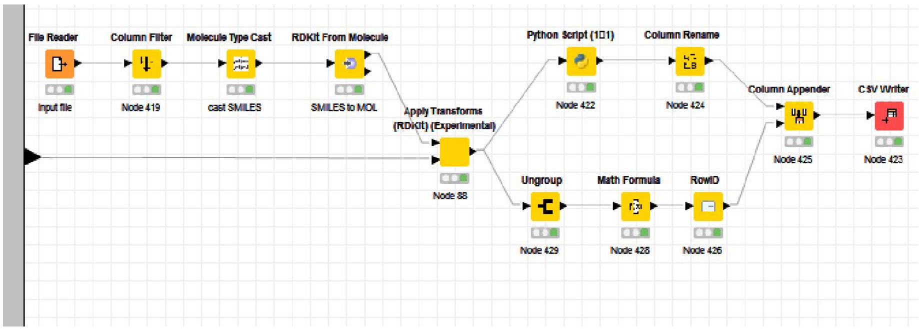

Figure 23. The constitute of MMP application and evaluation
