# Supplementary material for: Semi-automated workflow for molecular pair analysis and QSAR-assisted transformation space expansion
Source: J Cheminform. 2021 Nov 13;13:86. doi: 10.1186/s13321-021-00564-6 (PMC8590336; doi:10.1186/s13321-021-00564-6)
Supplement: Supplementary file 4 — Additional file 4: Figure S1. The comparison of (A) SD and (B) SEM of rules with different chemical clusters. Table S1. The introduction of the computational tools. [file 13321_2021_564_MOESM4_ESM.docx]

**Figure S1.** The comparison of (A) SD and (B) SEM of rules with different chemical clusters

**Table S1.** The introduction of the computational tools

| **Name** | **Description** |
| --- | --- |
| RDKit | Scaffold calculation, Descriptor/Fingerprint calculation and MMP chemical diversity calculation. |
| pandas | Python-data analysis |
| sklearn | Model construction |
| numpy | Python-n-dimensional arrays |
| matplotlib | Figure visualization |
| Scopy | Molecular pretreat and Descriptor/Fingerprint calculation |
| OpenBabel | Canonical SMILES and InCHIkey calculation |
